# Leukotriene B4 Is a Major Determinant of Leukocyte Recruitment During Otitis Media

**DOI:** 10.3389/fcimb.2021.768815

**Published:** 2021-12-22

**Authors:** Kyung Wook Heo, Kwang Pak, Arwa Kurabi, Allen F. Ryan

**Affiliations:** ^1^ Department of Otolaryngology, University Of California San Diego, La Jolla, CA, United States; ^2^ Department of Otorhinolaryngology - Head & Neck Surgery, Inje University Busan Paik Hospital, Busan, South Korea; ^3^ Research Section, Veterans Administration (VA) San Diego Healthcare System, La Jolla, CA, United States

**Keywords:** leukocytes, otitis media (OM), leukotriene B4 (LTB4), mucosa, non-typeable Haemophilus influenzae (NTHi), arachidonic acid (AA), inflammation

## Abstract

**Background:**

Pathogens of otitis media (OM) induce inflammatory responses in the middle ear (ME), characterized by mucosal hyperplasia, leukocyte infiltration, and inflammatory mediators, including arachidonic acid metabolites. We studied the role of the eicosanoid leukotriene B4 (LTB4) in OM.

**Methods:**

Expression of LTB4-related genes was evaluated by gene array and single-cell RNA-Seq in MEs infected with nontypeable *Haemophilus influenzae* (NTHi). An inhibitor of LTB4 receptor 1 (i.e. U75302) was also used to block LTB4 responses.

**Results:**

ME expression of LTB4-related genes was observed by gene arrays and scRNA-Seq. However, not all genes involved in LTB4 generation occurred in any one specific cell type. Moreover, LTB4 receptor inhibition significantly reduced mucosal hyperplasia and virtually eliminated leukocyte infiltration.

**Conclusions:**

ME expression of LTB4-related genes suggest a functional role in OM disease. The fact that LTB4-generation is spread across different cell types is consistent with a transcellular pathway of eicosanoid biosynthesis involving cell-to-cell signaling as well as transfer of biosynthetic intermediates between cells. The dramatic reduction in ME leukocyte infiltration caused by U75302 indicates that LTB4 plays a major role in ME inflammatory cell recruitment, acting *via* the LTB4R1 receptor. Given that there are many other chemotactic factors that occur in the ME during OM, the ability of LTB4 to activate leukocytes and stimulate their extravasation may explain the effects of inhibition. Reduction in mucosal hyperplasia due to U75302 administration may be secondary to the reduction in leukocytes since LTB4R1 is not expressed by mucosal epithelial or stromal cells. The results suggest that LTB4 receptor antagonists could be useful in treating OM.

## Introduction

Otitis media (OM) is the most prevalent disease of childhood (Casselbrant et al., 1993). More than 90% of children experience at least one episode of OM before the age of five. While the majority of acute OM is self-limiting and resolves within a few days even without treatment, 10-15% of children suffer from chronic or recurrent OM (Teele et al., 1989; Kaur et al., 2017). OM peaks between the ages of 6 months and two years (Pichichero, 2016), which is a critical period for the development of speech and language.

In developed countries, OM causes significant health and financial burdens. In the US, OM causes more pediatrician visits, antibiotic prescriptions, and surgeries than any other condition for children under 5 years of age (Rovers et al., 2008), at a cost estimated at $5 billion per year (Rosenfeld and Bluestone, 2003; Klein, 2000; Tong et al., 2018). Hearing loss due to chronic OM has been linked to delays in speech (Friel-Patti and Finitzo, 1993), language acquisition (Klausen et al., 2000), deficits in learning (Williams and Jacobs, 2009), and disorders of central auditory processing (Klein et al., 1988). However, in parts of the developing world, OM is a much more serious public health problem. The WHO estimates that undertreated OM, leading to chronic suppurative OM (CSOM), leads to 30,000 deaths per year due to meningitis. WHO also estimates that CSOM is responsible for half of the world’s burden of handicapping hearing loss, more than 275 million cases (WHO, 2004; Leach et al., 2020; WHO, 2020). It has even been suggested that CSOM represents a neglected tropical disease (Li et al., 2015).

OM is a multifactorial disease, with contributions from Eustachian tube dysfunction, prior upper respiratory viral infection, genetics, and environmental factors (Schilder et al., 2016). However, these factors often lead to a final common pathway of bacterial infection, primarily by nontypeable *Haemophilus influenzae* (NTHi), *Streptococcus pneumoniae* and/or *Moraxcela cattharalis*, with some viral co-infection (Ruohola et al., 2006). While streptococcal vaccines have reduced ME infections by covered strains, OM is an opportunistic infection and the incidence of ME infection by other species and streptococcal strains has increased (Casey et al., 2010). The result is that OM incidence has declined only modestly due to vaccinations (Vojtek et al., 2017).

Despite many years of research, treatments for OM have seen little improvement. Antibiotics remain the most common therapy for OM. Although antibiotics have been shown to be of little benefit for children over two, antibiotic prescriptions remain very common due largely to parental demand for treatment (McGrath et al., 2013). Pressure equalization tubes are recommended for chronic/recurrent OM, but they require surgery and general anesthesia for children, and can lead to scarring of the tympanic membrane (Yaman et al., 2010). Clearly, new therapies are desirable. Recent research on OM has illuminated pathways that contribute to OM pathogenesis and recovery (Kurabi et al., 2016). These pathways offer potential opportunities for alternative interventions.

The invading pathogens that are the hallmark of OM trigger innate and, eventually, adaptive immunity. Acute, uncomplicated OM typically resolves in a few days even without treatment (Rosenfeld and Kay, 2003). Because this is too soon for adaptive immune activation, it is clear that innate immunity is the default process for OM resolution. Many studies in animals and patients (see Kurabi et al., 2016 for review) have confirmed that innate immune receptors and effectors contribute to OM resolution. However, innate immune activation of inflammation also appears to be responsible for many of the pathogenic features of OM, including fluid infiltration of the ME producing serous OM, mucus generation leading to glue ear, and mucosal hyperplasia (Mittal et al., 2014). Understanding the roles of innate immune processes in OM may lead to the development of novel therapies.

A major component of inflammation is the recruitment of pro-inflammatory leukocytes, which invade the ME in large numbers in response to infection (Lim and Birck, 1971). Key among these pro-inflammatory cells are polymorphonuclear cells (PMNs) and monocytes (Qvarnberg et al., 1984), which enter the ME from the circulation in response to chemotactic factors. These can be secreted by macrophages that reside in the ME, or by non-immune ME cell types (Ryan et al., 2020). Monocytes that enter the ME typically differentiate into macrophages. PMNs and macrophages phagocytose and kill pathogens, but they also secrete pro-inflammatory substances that can harm middle ear tissues. Moreover, because of their relatively short life span in tissue (Colotta et al., 1992), dying PMNs release toxic substances such as the reactive oxygen species resident in their numerous intracellular granules as well as cellular debris that can impede clearance of ME fluid. The resultant inflammation induces fluid extravasation into the ME and hyperplasia of the ME mucosa, along with mucosal secretion of mucus and other factors.

**Figure 1:**
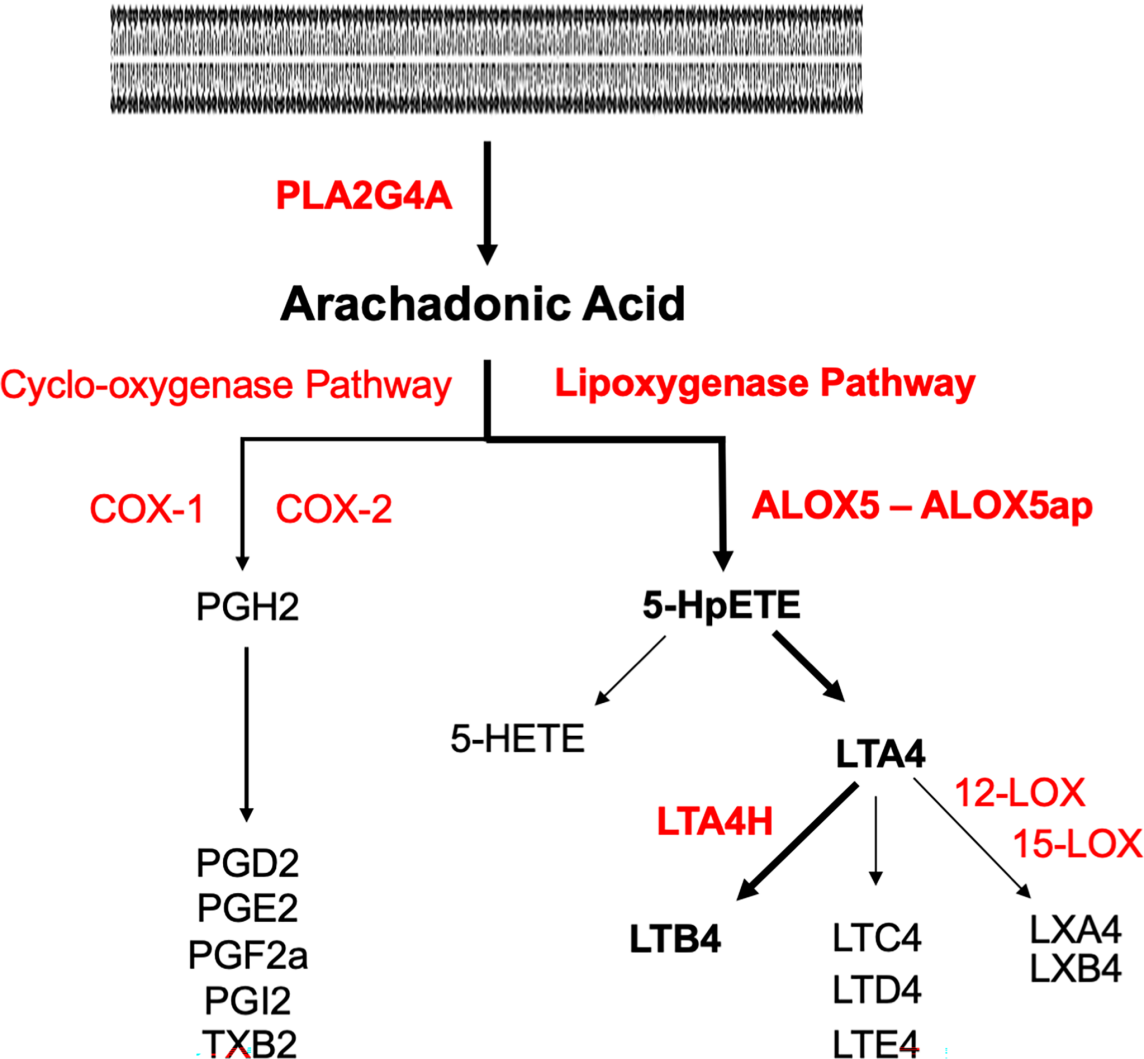
Pathways of arachidonic acid metabolism. Enzymes are in red, metabolites are in black. The pathway to LTB4 *via* the lipoxygenase pathway is delineated in bold. Phospholipase A2 (PLA2G4A) generates AA from cell membranes. Lipoxygenase A5 (ALOX5) and its activating protein (ALOX5AP) convert AA to 5HpETE, which then partitions between reduction to the 5-HETE and conversion to leukotriene A4 (LTA4). LTA4 hydrolase (LTA4H) converts LTA4 to LTB4.

Inflammation also induces arachidonic acid (AA) metabolism (Hanna and Hafez, 2018). As shown in **Figure 1**, the cytosolic enzyme phospholipase A2 group IV-A (PLA2G4A) converts cell membrane lipids into AA. AA can be metabolized by cyclo-oxygenases into prostaglandins, or by arachidonate-5-lipoxygenase (ALOX5) into the eicosanoid leukotriene A4 (LTA4). The latter process requires ALOX5 activating protein (ALOX5AP), which anchors ALOX5 to the membrane and plays an essential role in the transfer of AA to ALOX5. LTA4 in turn is converted by ALOX12 or ALOX15 into lipoxins. Alternatively, it can be converted by leukotriene A4 hydrolase (LTA4H) into leukotriene B4 (LTB4). The biological effects of leukotrienes and lipoxins often oppose each other, with LTB4 generally being pro-inflammatory and lipoxins being anti-inflammatory. LTB4 is a major chemoattractant of inflammatory leukocytes. LTB4 signals primarily *via* two LTB4 receptors, LTB4R1 and LTB4R2, which are expressed by leukocytes. LTB4R1 is a high-affinity receptor specific for LTB4, while LTB4R2 has a lower affinity and can also respond to other eicosanoids (Sheppe and Edelmann, 2021). Alternative, eicosanoid pathways lead to the production of other leukotrienes or lipoxygenases, while the cyclo-oxygenase pathway can generate lipoxygenases, prostaglandins, and thromboxane.

The role of LTB4 in OM is largely unexplored. The purpose of the present study was to evaluate the expression of genes associated with LTB4 generation as well as those encoding its receptors, during an acute episode of OM. Gene expression data are used because LTB4 is a lipid moiety with a half-life in minutes, and therefore difficult to quantify. To explore the functional role of LTB4 in OM, inhibition of the LTB4 receptor LTB4R1 was employed.

## Methods

### Animals

Mice used for gene array analysis were 60-90 day old C57BL/6:CB F1 hybrids, while those for single-cell RNA-Seq were C57BL/6 (Jackson Labs, Bar Harbor, ME, USA). Sprague-Dawley rats (Harlan Sprague Dawley, Indianapolis, IN, USA) were used for inhibitor studies. All experiments were performed to National Institutes of Health guidelines and approved by the VA San Diego Medical Center IACUC.

### OM Generation

Mice or rats were deeply anesthetized with rodent cocktail (ketamine 50 mg/kg, xylazine 1 mg/kg, acepromazine 5 mg/kg in 50 µl, i.p.) and the ME bulla surgically exposed. A small opening was created in each bulla and *Haemophilus influenzae* strain 3655 (nontypeable, biotype II; NTHi), was infected in 5 μl PBS for mice and 50 μl for rats, at a titer of 10^4^.

### Gene Array

Gene arrays were used to provide quantitative information on gene expression levels within the ME, since mRNA is extracted in a uniform manner from all cells in a tissue. Forty mice per time point were inoculated in the ME bilaterally with NTHi. Uninoculated animals served as controls. Mucosal tissue and exudate were harvested from 20 mice at each of the following intervals: 0 hours (0h, no treatment), 3h, 6h, 24h, 2 days (2d), 3d, 5d and 7d after inoculation, and pooled. The tissue was homogenized in TRIzol (Life Technologies, Carlsbad, CA) and total RNA extracted, reverse transcribed, amplified and transcribed *in vitro* to generate biotinylated cRNA probes that were hybridized to 2 Affymetrix (Agilent Technologies, Santa Clara, CA) MU430 2.0 microarrays, according to the manufacturer’s protocol. This procedure was duplicated for each time point to obtain a second, independent biological replicate. Thus each data point represents 2 separate samples consisting of 20 mice each, and 4 Affymetrix arrays. Specific genes were assessed at individual time points using Genespring GX 7.3 (Agilent Technologies, Santa Clara, CA). To identify changes in gene expression, the data were first analyzed using a variance modeling approach. The raw MAS5 expression values were imported into the VAMPIRE software without prior normalization. This program uses a Bayesian approach to identify significantly altered genes (Hsiao et al., 2005). Hybridization to probes for genes involved in AA metabolism and signaling were assessed at individual time points for difference from control (uninfected) samples, after Bonferonni correction for multiple tests and using ANOVA in Genespring GX 7.3 (Agilent Technologies, Santa Clara, CA). Additional details of methods are provided in our previous publication (Hernandez et al., 2015) in which the genes evaluated here were not included.

### Single-Cell RNA-Seq

Gene arrays generated from bulk tissue cannot identify which cells are expressing a given gene. We used single-cell RNA-Seq (scRNA-Seq) to provide precise, cell-level data. However, because the isolation of different cell types will vary in efficiency depending upon fragility and strength of bonding to other cells, this method is less able to determine overall ME levels of gene expression. Groups of six C57BL/6 mice each were untreated as controls. Additional groups were inoculated in the ME with NTHi and mucosal tissue and exudate harvested 24 hours later, when the gene array data indicated maximum regulation of genes related to LTB4. The pooled tissue for each sample was digested with thermolysin (0.5 mg/ml, Sigma-Aldrich, #T7902) followed by FACSMax cell dissociation solution (Genlantis, #T200100), and triturated into single cells. Dissociated cells were diluted to 700 cells/μL. Three replicates were performed to obtain independent biological samples of control MEs, while four replicates were performed 24 hours after NTHi inoculation. Single-cell libraries were prepared using the Chromium Controller (10X Genomics, Pleasanton, CA) according to the manufacturers’ instructions. The libraries were sequenced on an Illumina HiSeq 2500 (Illumina, San Diego) and yielded approximately 200 million reads per sample.

Bar-coded reads were demultiplexed using Cellranger 2.0.2 (10X Genomics) and mkfastq in conjunction with bcl2fastq 2.17.1.14 (Illumina) and aligned to a murine reference genome mm10 (Ensembl 93) provided by 10X Genomics. Reads were filtered to remove short reads and reference genome mismatches to improve library quality, quantified and subjected to principal component analysis (PCA) clustering. For the three samples from normal MEs, the average number of analyzed cells/sample was 2,257, and the number of genes detected/sample averaged 17,323. For the four samples at 24 hours the number of cells/sample averaged 3,978 and genes detected averaged 17,413. Identification of cells in each cluster was based on the following marker genes: epithelial cells, high expression of *Epcam and Krt18*; ciliated epithelial cells, *Epcam* and *Hydin*; immature epithelial cells, low *Epcam and Krt18*; stromal cells, *Col1a2*; vascular endothelial cells, *Egfl7* and *Flt4*; lymphatic endothelial cells *Egfl7* and *Flt1*; pericytes, *Rgs5*; monocytes, *Csf1r*; lymphocytes, *Ptprcap*; and melanocytes, *Mlana*. After infection, PMNs were identified by expression of *Il1f9* and *Stfa2l1* and RBCs by *Hba-a1*. Graph-based and K-means analysis of gene expression was then performed for genes related to LTB4 generation and sensing. Expression of genes by ME cells was visualized using 10X Genomics cLoupe. A cluster plot for each gene was generated using log2 data, for optimal visualization of expressing cells. Violin plots of expression levels for each cluster were log-normalized for optimal comparison across the cluster cell population. Additional details of methods are available in our previous publication on normal ME scRNA-Seq (Ryan et al., 2020), in which no genes related to AA metabolism were reported. The gene data is available at the Science Data Bank (https://www.scidb.cn/en) under doi:10.11922/sciencedb.01353.

### Inhibitor Studies

Because of the small size (5 μL) of the mouse ME, inhibitor studies were performed in rats. Rats and mice are closely related and have highly similar responses to ME NTHi inoculation (Melhus et al., 1994; Melhus and Ryan, 2003). For the inhibitor study, 4 rat MEs per treatment were inoculated with NTHi as above. Control rats received NTHi alone in PBS plus 0.1% DMSO. Inhibitor groups received NTHi with different doses of the specific BLT1 inhibitor U75302 [(6-​(6-​(3R-​hydroxy-​1E,​5Z-​undecadien-​1-​yl)-​2-​pyridinyl)-​1,​5S-​hexanediol; refs; Cayman Chemical Company, Ann Arbor, MI, USA] plus DMSO. Three U75302 dosages were used: 1.5 μM, 15 μM, 150 μM. 150 μM U75302 only into the ME served as an additional control. Rats were sacrificed for ME harvest 48 hours after inoculation.

### Histology

Rats were sacrificed by decapitation under deep anesthesia. ME bullae were extracted, post-fixed with 4% PFA overnight, and decalcified in an 8% EDTA/4% PFA solution over a 14-day period. The bullae were embedded in paraffin and sectioned at 7 μm. Sections were stained with hematoxylin-eosin and mounted. As described previously (Leichtle et al., 2010), from four sections at the midpoint of each ME, mucosal thickness was measured at six standard locations and averaged to obtain a measure for that ME. From the same sections, the total area of the ME lumen and the percent of the lumen area occupied by leukocytes were digitally determined using NIH Image software. A 300X image of the ME infiltrate was captured for each section, and the number of neutrophils and macrophages quantified to capture the proportions of each cell type in the infiltrate. Leukocyte data were not normally distributed and were analyzed using the Kruskal-Wallace nonparametric ANOVA.

## Results

### Gene Array

**Figure 2:**
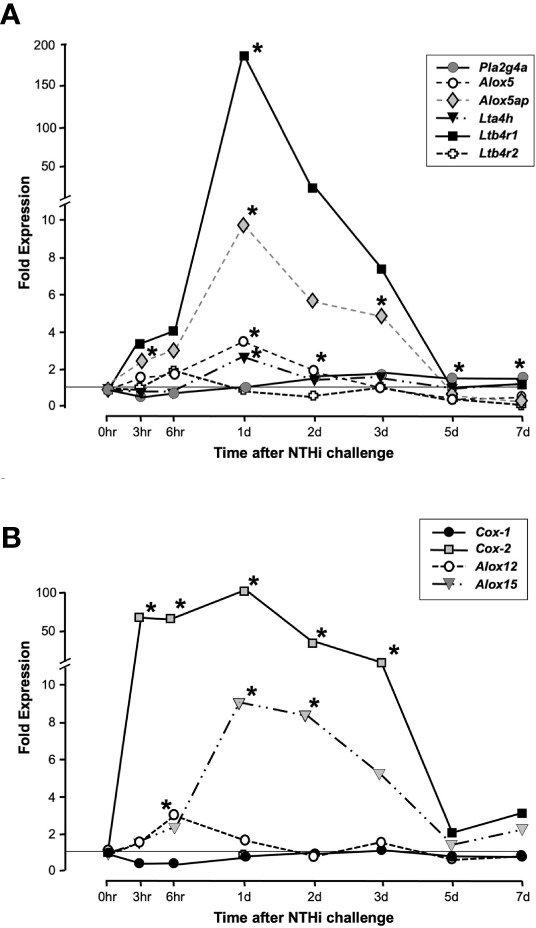
Expression of genes involved in LTB4 generation and sensing during a complete episode of acute NTHi-induced OM in the mouse, from initiation to recovery, evaluated by gene arrays. Panel **(A)** shows AA Lipoxygenase pathway related genes. *Ltb4r1* is very strongly and significantly upregulated during OM, with lesser but significant upregulation of *Alox5*, *Alox5ap* and *Lta4h*. *Ltb4r2* was not significantly regulated. Panel **(B)** shows related enzymes of AA metabolism. *and gray color = p <.05, ANOVA. Detailed data on fold change ranges and p values are presented in the **Supplementary Table**.

As assessed by cRNA probe hybridization to gene arrays, several genes involved in LTB4 production and sensing were significantly upregulated following NTHi inoculation of the ME (**Figure 2A**). The *Pla2g4a* gene was only slightly (1.5-fold) upregulated during OM. The *Alox5* gene increased expression 3-fold at 1 day, while *Alox5ap* increased expression 5-fold at 3 days after inoculation. The *Lta4h* gene for the LTB4 generating LTA4 hydrolase enzyme increased 2-fold at 1 day. Most dramatically, the LTB4 receptor gene *Ltb4r1* increased by 170-fold at 1 day after NTHi inoculation of the ME, while *Ltb4r2* was not significantly regulated. Some other enzymes of AA metabolism were also significantly regulated, especially *Cox2* but also *Alox12* and *Alox15* (**Figure 2B**). Detailed data on fold change ranges and statistics are presented in the **Supplementary Table**.

### Single-Cell Gene Expression

ScRNA-Seq was used to map the expression of LTB4 synthesis and sensing genes to uninfected ME cells, and cells harvested at 24 hours after NTHi inoculation when expression of most genes peaked (**Figure 2A**). PCA generated 10-15 clusters for each condition, which corresponded to different ME cell types. Some cell types, such as epithelial cells, consisted of multiple PCA clusters that corresponded to recognized subtypes. Other cell types, such as PMNs, consisted of several clusters not easily defined. After identification of the cell types in each cluster, genes related to LTB4 generation and sensing were visualized using the 10X Genomics cLoupe function.

**Figure 3:**
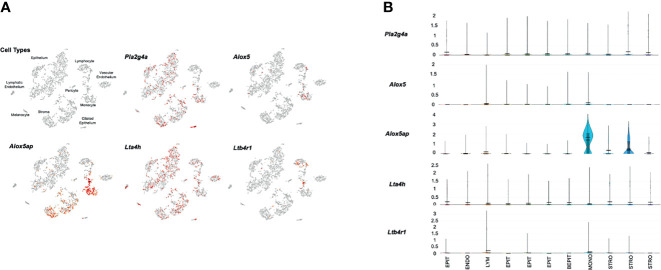
Expression of LTB4-related genes by individual ME cell types, before inoculation of the murine ME with NTHi. For each sample, twelve MEs were pooled and digested into a single-cell suspension for single-cell RNA-Seq. Panel **(A)** represents 2,858 cells recovered from the normal ME, clustered by principal component analysis, and identified by recognized cell-type marker genes. Expression of each gene by cell is indicated in red. Panel **(B)** presents violin plots for individual PCA clusters, labeled by cell type, with some cell types represented by multiple clusters. The width of each plot represents the frequency of cells expressing the gene at that log normalized level. The upper horizontal line in each plot represents the cluster mean, and the lower line the median. The height of the vertical line represents the highest expressing cell.

**Figure 3** presents gene expression by cells in the uninfected ME. The expression by cell types is illustrated in the cLoupe PCA projection of **Figure 3A**, which shows the results in a representative control sample. As can be seen in the figure, *Pla2g4a* mRNA was expressed by a small number of most ME cell types, with the exception of ciliated epithelial cells, where most cells were positive, and endothelial cells, melanocytes and lymphocytes, which were essentially negative. *Alox5* was observed in small subsets of monocytes and lymphocytes. *Alox5ap* was expressed in most monocytes and approximately half of the stromal cells, as well as a few epithelial cells and lymphocytes. *Lta4h* was observed in small subsets of all ME cell types, while *Ltb4r1* was prevalent only in subsets of monocytes and lymphocytes. *Ltb4r2* was not expressed by any ME cells. **Figure 3B** shows violin plots of gene expression by PCA cluster, from the same control sample. The plots provide a relative quantitative analysis of gene expression. They confirm that only a small number of cells expressed LTB4-related genes at high levels, with the majority of cells not expressing. The exceptions were monocytes and stromal cells, of which many cells expressed *Alox5ap* at appreciable levels.

**Figure 4:**
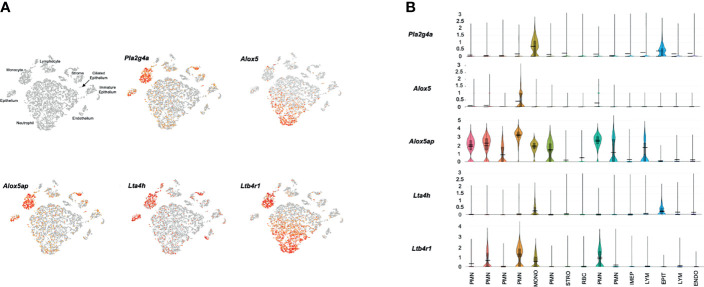
Expression of LTB4-related genes by individual ME cell types, 24 hours after inoculation of the murine ME with NTHi. **(A)** PCA clusters of 9,604 cells, with cell types identified and cells expressing each gene indicted in red. **(B)** Violin plots demonstrating cell type expression patterns as well as the much higher level expression of LTB4-related genes.

**Figure 4** presents results from a representative ME sample collected 24 hours after NTHi infection. All of the cell types observed in control MEs were present, except for melanocytes. In addition, a new category of immature epithelial cells was present, and large numbers of neutrophils and a few erythrocytes had infiltrated the ME. In agreement with our array data, substantially more expression of LTB4-related genes was observed 1 day after infection. *Pla2g4a* mRNA was expressed in virtually all monocytes and variable subsets of other cell types. *Alox5* mRNA was observed primarily in a subset of infiltrating neutrophils, and a few monocytes. *Alox5ap* was expressed by almost all monocytes, the majority of neutrophils and small subsets of other cell types. *Lta4h* was present in most epithelial cells and monocytes, but also by small subsets of other cell types. *Ltb4r1* was produced exclusively by monocytes and a subset of neutrophils. *Ltb4r2* expression again was not observed. The violin plots of **Figure 4B** confirm the distribution of LTB4-related gene expression by cell type, and provide relative quantitative expression levels which can be compared to those for the cell types in **Figure 3B**. Each of the genes was differentially expressed at a significant level (p<.05, ANOVA) between control and infected MEs, for the cell types with major differences in violin plots. PMNs, which were not present in the control sample, could not be thus analyzed.

### Mucosal Hyperplasia

**Figure 5:**
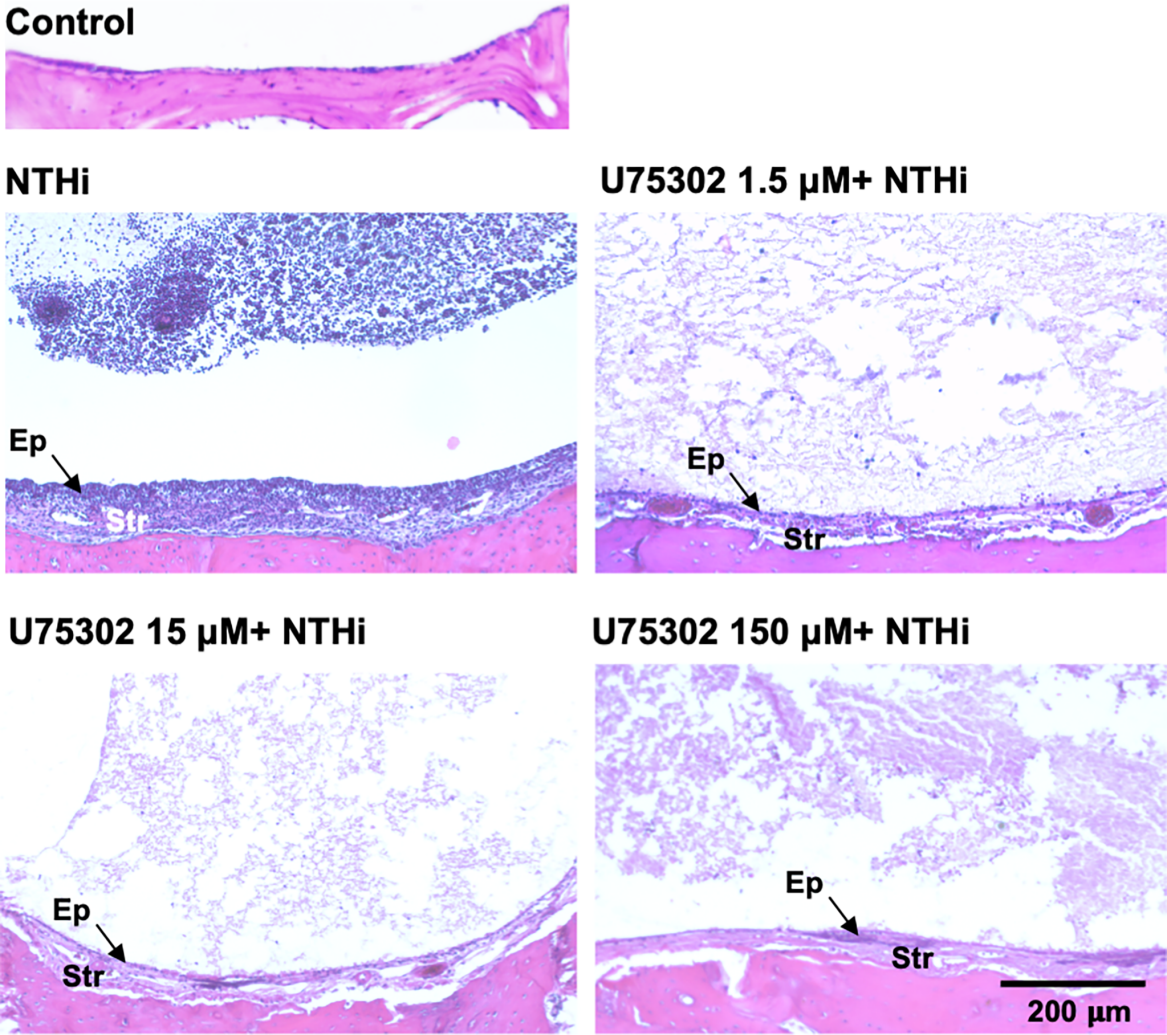
The MEs of a control, untreated mouse and of mice 2 days after infection with NTHi, or NTHi plus different doses of the specific LTB4R1 inhibitor U75302. At all doses, hyperplasia of both the mucosal epithelium (Ep) and stromal (Str) was reduced. In addition, infiltration of the ME lumen by leukocytes was virtually eliminated. A luminal precipitate was observed in all treated ears, which presumably represents an acellular exudate.

**Figure 6:**
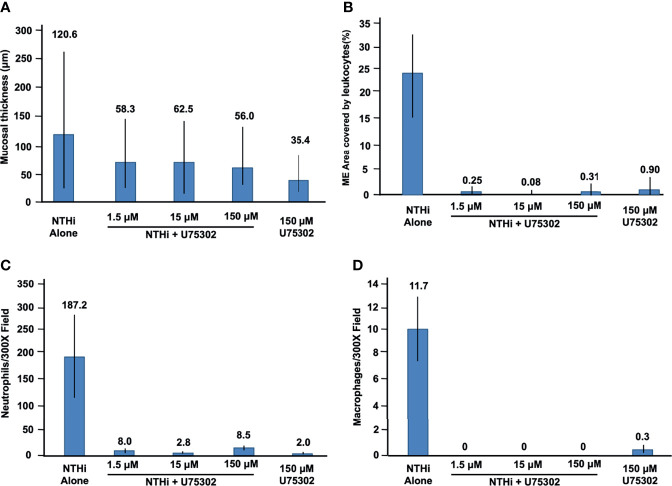
Quantitative evaluation of the effects of U75302 on OM. **(A)** ME mucosal thickness (mean, range: max and min). **(B)** Percent of the ME lumen occupied by leukocytes. **(C)** Number of neutrophils counted in high-power images of ME exudate. **(D)** Number of macrophages counted in high-power images of ME exudate.

**Figures 5** shows representative examples of ME histology for control MEs, before infection, for MEs 48 hours after inoculation with NTHi alone, for MEs after 48 hours after inoculation with NTHi plus one of three dosages of U75302, and in MEs 72 hours after injection of the highest dosage of U75302 alone. Quantitative analysis of mucosal thickness is provided in **Figure 6A**. ME infection with NTHi substantially increased mucosal thickness when compared to control MEs. All three dosages of U75302 significantly reduced mucosal thickness (p <.05; ANOVA). Thickness after injection of U75302 alone was not significantly different from control.

### Leukocyte Infiltration of the ME

**Figure 5** also illustrates the leukocyte response of the ME to NTHI inoculation, with and without the LTB4R1 inhibitor U75302. It is clear from **Figure 5** that leukocyte infiltration of the ME lumen was dramatically reduced by all three dosages of inhibitor. This is confirmed by the quantitative analysis presented in **Figure 6B** (p <.001; Kruskal Wallis ANOVA). However, the presence of substantial amounts of precipitate in the ME suggests that LTB4 inhibition did not reduce infiltration of the ME by fluid from the vasculature. U75302 alone did not induce significant cellular or fluid infiltration of the ME.

### Leukocyte Cell Types in the ME

Infection by NTHi typically produces ME infiltration by large numbers of PMNs, and a lower number of monocytes/macrophages. This was also observed in the control animals of the present study. **Figures 6C, D** represent each cell type as counted in 300X micrographs of infiltrate in the ME lumen. Not surprisingly given the data shown in **Figure 6B**, treatment with U75302 virtually eliminated both cell types from the ME (p <.001; Kruskal Wallis ANOVA).

## Discussion

Significant increases in the expression of genes related to LTB4 generation and receptor sensing after ME inoculation with NTHi provide evidence that this AA metabolite could play a role in OM. In particular, the dramatic rise in *Ltb4r1* mRNA observed early in OM suggests that either ME cells generated large amounts of this receptor, or that cells expressing these receptors entered the ME in large numbers. Conversely there were no significant changes in the expression of *Ltb4r2 mRNA*. Genes involved in the generation of LTB4 also peaked at 24 hours, with *Alox5ap* also showing a significant increase earlier. Infection also increased the expression of other genes of the AA metabolic pathway, in both the cyclo-oxygenase and lipoxygenase pathways. These data suggest that other AA metabolites participate in OM, and their production has been documented in experimental OM (Goldie et al., 1993). However, responses to any of these would not be expected to be inhibited by U75302.

Single-cell RNA-Seq confirms and validates the expression of mRNA encoding components of the LTB4 synthetic pathway, and its specific receptor LTB4R1. Interestingly, all of the synthetic components were not present in any one ME cell type, but rather were distributed across a variety of cells. This is consistent with other cellular systems, where eicosanoids are often the result of intercellular interactions involving cell-to-cell signaling and the transfer of biosynthetic intermediates, such as LTA4, between cells (e.g. Fradin et al., 1989). This process is known as the transcellular pathway of eicosanoid biosynthesis (Sala et al., 2010). It requires one cell to synthesize and release a component of the biosynthetic pathway, and another cell to take up that intermediate and process it into a final active molecule. In the case of eicosanoids, this is aided by their lipid solubility.

This intercellular process appears to occur in the ME as well. From the pattern of *Pla2g4a* expression, it can be inferred that most AA is produced by ME epithelial cells, with a contribution from some resident leukocytes. The enzymes that produce LTB4 are primarily expressed by leukocytes, but the production of its precursor, LTA4H, is mostly by epithelial and stromal cells. The exchange of AA and LTA4H between epithelial and leukocytic cell types, and potentially signaling between the cell classes, appears to be necessary for the ME production of LTB4. The expression of LTB4R1 only in leukocytes was expected. The absence of cells expressing mRNA encoding the specific LTB4 receptor LTB4R2 indicates that inhibition of LTB4R1 would completely block LTB4 signaling in the ME.

The final step in LTB4 production involves the conversion of LTA4 to LTB4 by LTA4H (see **Figure 1**). *Lta4h* mRNA is highly expressed in epithelial cells and monocytes, and some stromal cells, vascular endothelial cells, and PMNs. These cells are likely to be the primary sources of direct LTB4 production. However, PMNs produce the most ALOX5 and are likely the primary source of the precursor molecules 5-HpETE and LTA4.

The virtual elimination of leukocytic infiltration of the ME caused by the LTB4R1 inhibitor U75302 indicates that LTB4 activation of this receptor is required for the recruitment of PMNs and monocytes/macrophages to the tympanic cavity. Given that several other chemoattractants that target these two cell types are also expressed in the ME (Hernandez et al., 2015; Dennifel et al., 2017; Hur et al., 2021), the dominant role of LTB4 was unexpected. It seems likely that LTB4’s role in the activation of leukocytes, which is required for extravasation, is responsible for its ME effects since this could disable recruitment by all chemoattractants.

The results of our animal study have potential clinical implications. A prior study found that LTB4 is present in ME fluid from children, and that high levels are associated with treatment failure (Chonmaitree et al., 1996). The authors concluded that LTB4 may play role in delaying acute OM recovery and recurrence. They suggested that more effective treatment of OM would require the combined use of antibiotics that reduce inflammatory leukocyte function. Another study from the same group found that treatment with steroids did not decrease LTB4 in the ME fluid of children (McCormick et al., 2003). Our results indicate that LTB4 receptor antagonists, or perhaps natural pro-resolution compounds that oppose the effects of LTB4, such as resolvin E1 (Arita et al., 2005), may be useful in treating OM.

## Data Availability Statement

The data presented in the study are deposited in the Science Data Bank repository, accession number 31253.11.sciencedb.01353 and can be viewed at https://www.scidb.cn/en/s/BJJBBv.

## Ethics Statement

The animal study was reviewed and approved by VA Health IACUC.

## Author Contributions

KH and KP performed experiments and data collection. AR, KH, and AK discussed the study idea, analyzed the data and generated the figures. KH wrote the initial manuscript draft. All authors contributed to final manuscript.

## Funding

Supported by grants DC006279, DC000129, and DC012595 from the NIH/NIDCD and BX001205 from the VA.

## Conflict of Interest

AR is a co-founder of Otonomy Inc., serves as a member of the Scientific Advisory Board, and holds an equity position in the company. The UCSD Committee on Conflict of Interest has approved this relationship. Otonomy, Inc. played no part in the research reported here.

The remaining authors declare that the research was conducted in the absence of any commercial or financial relationships that could be construed as a potential conflict of interest.

## Publisher’s Note

All claims expressed in this article are solely those of the authors and do not necessarily represent those of their affiliated organizations, or those of the publisher, the editors and the reviewers. Any product that may be evaluated in this article, or claim that may be made by its manufacturer, is not guaranteed or endorsed by the publisher.
