# Supplementary material for: Leukotriene B4 Is a Major Determinant of Leukocyte Recruitment During Otitis Media
Source: Front Cell Infect Microbiol. 2021 Dec 22;11:768815. doi: 10.3389/fcimb.2021.768815 (PMC8727869; doi:10.3389/fcimb.2021.768815)
Supplement: Supplementary file 1 [file Table_1.doc]

**SUPPLEMENTARY TABLE**

**Time Fold change (range) P value**

***Pla2g4a* 1448558_a_at**

Time 0h 0.992 (0.874 to 1.126) 0.960

Time 3h 0.691 (0.607 to 0.788) 0.216

Time 6h 0.708 (0.480 to 1.045) 0.538

Time 1d 1.089 (0.938 to 1.264) 0.670

Time 2d 1.435 (1.390 to 1.482) 0.056

Time 3d 1.552 (1.297 to 1.857) 0.247

Time 5d 1.326 (1.316 to 1.335) 0.017

Time 7d 1.488 (1.479 to 1.497) 0.009

***Alox5* 1441962_at**

Time 0h 0.987 (0.800 to 1.160) 0.949

Time 3h 1.431 (1.342 to 1.525) 0.113

Time 6h 1.640 (1.299 to 2.071) 0.280

Time 1d 3.236 (3.110 to 3.367) 0.022

Time 2d 1.623 (1.616 to 1.629) 0.005

Time 3d 1.261 (1.068 to 1.490) 0.396

Time 5d 0.497 (0.418 to 0.590) 0.154

Time 7d 0.522 (0.375 to 0.726) 0.299

***Alox5ap* 1452016_at**

Time 0h 1.000 (0.998 to 1.002) 0.999

Time 3h 2.442 (2.346 to 2.541) 0.029

Time 6h 3.163 (3.090 to 3.238) 0.013

Time 1d 9.598 (7.932 to 11.61) 0.054

Time 2d 5.494 (4.225 to 7.144) 0.097

Time 3d 4.609 (4.577 to 4.641) 0.003

Time 5d 1.235 (1.121 to 1.360) 0.273

Time 7d 1.420 (1.139 to 1.771) 0.357

***Lta4h* 1453528_at**

Time 0h 0.999 (0.946 to 1.054) 0.983

Time 3h 0.834 (0.769 to 0.904) 0.266

Time 6h 0.719 (0.599 to 0.864) 0.323

Time 1d 2.373 (2.245 to 2.508) 0.041

Time 2d 1.748 (1.278 to 2.392) 0.326

Time 3d 1.771 (1.713 to 1.831) 0.037

Time 5d 1.300 (1.172 to 1.442) 0.239

Time 7d 1.294 (1.137 to 1.473) 0.297

***Ltb4r1* 142047_at**

Time 0h 0.929 (0.630 to 1.370) 0.881

Time 3h 3.177 (2.247 to 4.492) 0.185

Time 6h 3.930 (2.978 to 5.188) 0.127

Time 1d 162.9 (135.7 to 195.0) 0.023

Time 2d 32.92 (20.87 to 51.40) 0.083

Time 3d 6.783 (2.477 to 18.57) 0.308

Time 5d 0.982 (0.768 to 1.256) 0.954

Time 7d 1.266 (0.939 to 1.707) 0.575

***Ltb4r2* 145080at**

Time 0h 0.872 (0.511 to 1.489) 0.841

Time 3h 0.890 (0.603 to 1.313) 0.814

Time 6h 2.358 (1.856 to 2.998) 0.173

Time 1d 1.316 (1.008 to 1.718) 0.490

Time 2d 0.847 (0.327 to 2.194) 0.890

Time 3d 1.764 (1.304 to 2.386) 0.311

Time 5d 0.766 (0.659 to 0.890) 0.327

Time 7d 0.381 (0.364 to 0.398) 0.030

***Cox-1* 1436448_a_at**

Time 0h 0.984 (0.822 to 1.178) 0.943

Time 3h 0.525 (0.476 to 0.578) 0.095

Time 6h 0.569 (0.383 to 0.846) 0.390

Time 1d 0.740 (0.702 to 0.781) 0.112

Time 2d 0.874 (0.774 to 0.986) 0.465

Time 3d 1.182 (1.053 to 1.326) 0.385

Time 5d 0.697 (0.666 to 0.729) 0.0795

Time 7d 0.634 (0.544 to 0.740) 0.208

***Cox-2* 1417262_at**

Time 0h 0.980 (0.802 to 1.198) 0.937

Time 3h 66.67 (49.46 to 89.86) 0.0452

Time 6h 63.12 (61.59 to 64.69) 0.00376

Time 1d 104.8 (74.92 to 146.5) 0.0458

Time 2d 38.00 (28.69 to 50.32) 0.0491

Time 3d 18.78 (15.43 to 22.86) 0.0426

Time 5d 2.035 (1.458 to 2.842) 0.28

Time 7d 2.902 (1.706 to 4.937) 0.294

***Alox12* 1422700_at**

Time 0h 1.000 (0.947 to 1.053) 0.983

Time 3h 1.590 (1.325 to 1.895) 0.236

Time 6h 3.150 (2.957 to 3.359) 0.0353

Time 1d 1.670 (1.540 to 1.800) 0.0966

Time 2d 0.740 (0.628 to 0.878) 0.327

Time 3d 1.540 (1.348 to 1.763) 0.192

Time 5d 0.78 (0.655 to 0.928) 0.389

Time 7d 1.31 (0.933 to 1.830) 0.573

***Alox15*** **1420338_at**

Time 0h 0.963 (0.731 to 1.269) 0.914

Time 3h 1.564 (1.510 to 1.621) 0.0504

Time 6h 2.308 (2.027 to 2.628) 0.0981

Time 1d 8.997 (7.620 to 10.62) 0.048

Time 2d 8.149 (7.12 to 9.328) 0.0409

Time 3d 4.728 (3.583 to 6.238) 0.112

Time 5d 1.488 (1.227 to 1.804) 0.288

Time 7d 1.886 (1.81 to 1.965) 0.041
